# A Sensing and Tracking Algorithm for Multiple Frequency Line Components in Underwater Acoustic Signals

**DOI:** 10.3390/s19224866

**Published:** 2019-11-08

**Authors:** Xinwei Luo, Zihan Shen

**Affiliations:** Key Laboratory of Underwater Acoustic Signal Processing of Ministry of Education, Southeast University, Nanjing 210096, China; 220180802@seu.edu.cn

**Keywords:** frequency line detection, lofargram, HMM, lofargram segmentation

## Abstract

Reliable and efficient sensing and tracking of multiple weak or time-varying frequency line components in underwater acoustic signals is the topic of this paper. We propose a method for automatic detection and tracking of multiple frequency lines in lofargram based on hidden Markov model (HMM). Instead of being directly subjected to frequency line tracking, the whole lofargram is first segmented into several sub-lofargrams. Then, the sub-lofargrams suspected to contain frequency lines are screened. In these sub-lofargrams, the HMM-based method is used for detection of multiple frequency lines. Using image stitching and statistical model method, the frequency lines with overlapping parts detected by different sub-lofargrams are merged to obtain the final detection results. The method can effectively detect multiple time-varying frequency lines of underwater acoustic signals while ensuring the performance under the condition of low signal-to-noise ratio (SNR). It can be concluded that the proposed algorithm can provide better multiple frequency lines sensing ability while greatly reducing the amount of calculations and providing potential techniques for feature sensing and tracking processing of unattended equipment such as sonar buoys and submerged buoys.

## 1. Introduction

**Figure 1:**
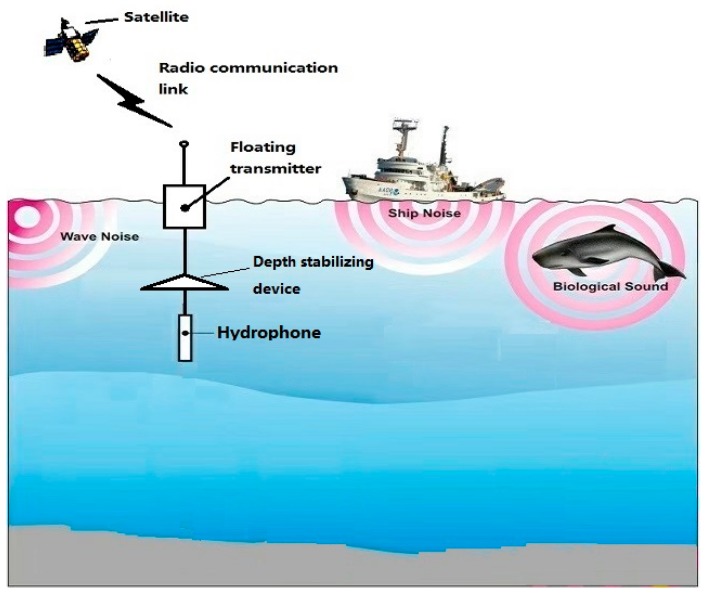
Schematic diagram of buoy and submerged buoy system.

The underwater acoustic signals in the ocean contain marine environmental noise, radiated noise from surrounding ships, and signals from various sonars. In order to obtain target information from the ocean, a convenient way is to deploy sensors such as buoys and submerged buoys in the sea of interest and analyze the received underwater acoustic signals to achieve observations of nearby sea areas. Due to the simple structure of the buoy and the submarine system, the resolving power of different azimuth targets is often lacking. Therefore, sensing and tracking the narrow-band line spectral components associated with the target in the underwater acoustic signal is one of the primary tasks. There are several problems when using the unmanned platform such as buoy or submerged buoy to acquire the target signal. As shown in Figure 1, the hydroacoustic signal components in the ocean are complex and contain environmental noise, target radiated noise, and target sonar signals. The feature extraction algorithm needs to have better tolerance to the signal-to-noise ratio and signal form to extract more signal features; Secondly, because of the size and power consumption limitations of unmanned platforms, algorithms need to have higher processing efficiency. Therefore, the narrowband signal sensing and extraction algorithms of the buoy and submerged buoy system should have the following characteristics: (1) autonomy; (2) wide adaptability; and (3) high efficiency.

Traditional approaches for frequency line tracking have been based on alpha-beta or Kalman filter-based trackers. When performing frequency line tracking, it is usually based on nearest neighbor logic. When using such methods for frequency line tracking, the latter half usually needs to filter the most suitable frequency points through nearest neighbor clustering. That is to say, the detected frequency line consists of the frequency points with the highest signal-to-noise ratio (SNR) close to the real frequency line. For example, the literature [1,2] first performs outlier point detection on the data of each time frame in the lofargram, and then clusters the detected outlier points from the time dimension. Under certain conditions of SNR, these methods can effectively detect and track the frequency line. While the above methods cover the mechanism for state estimation in the tracker, they do not describe the data association logic. Frequency line tracking can also be performed using methods based on image processing [3]. The method based on image processing mainly treats the lofargram as an image and transforms the extraction of the frequency lines into the detection of the edge information in the image. Abel et al. [4] proposed a frequency line detection method based on image processing and machine vision. Gillespie [5] first smooths the spectrogram using a Gaussian filter and then extracts the frequency line information using an edge detection algorithm. In the literature [6], lofargram is firstly denoised by nonlinear enhancement and convolution denoising algorithms, and then the frequency lines are extracted from lofargram using the characteristics of frequency lines. In the case of low SNR, the frequency line information is likely to be lost during the image conversion. Therefore, the effect of frequency line extraction of the above method based on image processing may be deteriorated when the SNR is low.

Many other data association methods are available, particularly for dealing with multiple targets [7]. These include assignment algorithms, joint probabilistic data association (JPDA) [8], multiple hypothesis tracking (MHT) [9] and Viterbi data association [10]. All of these methods assume that the state space is continuous and therefore a Kalman filter can be used to provide the state estimates. In the case of nonlinear dynamics, various filtering approaches can be applied, such as extended Kalman filters and Gaussian mixtures. More modern variants include unscented Kalman filters and particle filters. In contrast to frequency line tracking approaches that utilize a continuous state space, a different class of approaches is possible for discrete state spaces. In this case, the system of ordinary differential equations that models the time evolution of the target’s dynamical state is replaced by a description based on hidden Markov model (HMM). Streit and Barrett [11] proposed a method for frequency line tracking in the lofargram using HMM, however this method is only applicable to the case where a single frequency line trace exists. Barrett and Holdsworth [12] combined the amplitude and phase information of the signal spectrum to present a more accurate frequency line tracking method. Van and Alinat [13] extended the method based on the research of Streit and Barrett [11], and presented a multi-frequency line detection model based on multi-model detection method. Paris and Jauffret [14,15] elaborated on the method of multi-frequency line tracking using forward-backward (FB) algorithm and Viterbi algorithm, and gave the mathematical model of the known and unknown SNR. Based on the work of the predecessors, Pulford and Tyson [16] proposed a HMM tracking method based on one-dimensional data, which greatly improves the efficiency of frequency line extraction. Although in the actual products of radar, sonar and video processing systems, the approaches that utilise a continuous state space are widely used. However, HMM-based frequency line tracking method has better performance under low SNR conditions and is suitable for tracking at very low detection thresholds or even on unthresholded data. This advantage of the HMM-based approach is of great significance for frequency line tracking in passive sonar signals.

The proposed method for detecting multiple frequency lines in lofargram is mainly based on HMM. Although the literature [13,14,15,16] can effectively track frequency lines, the calculation of these methods is still huge, and it is difficult to adapt to the analysis of data with a large number of frequency lines. The sensing and tracking algorithm for multiple frequency line components in underwater acoustic signals proposed in this paper is divided into four steps: (1) data preprocessing, (2) lofargram segmentation and sub-lofargram screening, (3) HMM-based frequency line tracking, and (4) merging overlapping frequency lines in different sub-lofargrams. In step 1, the lofargram of the signal is obtained by time-frequency analysis and background equalization. In step 2, the whole lofargram is segmented into several sub-lofargrams and the sub-lofargrams with frequency lines are screened out by statistical analysis of each sub-lofargram. This step can reduce the number of frequency states at each iteration while ensuring the continuity of frequency states between different sub-lofargrams, thus greatly reducing the complexity of HMM-based frequency line detection methods. In step 3, the parameters in HMM are set according to prior information and data in sub-lofargram. Then the frequency lines in each sub-lofargram are extracted sequentially using HMM-based method. In step 4, the accurate frequency lines are obtained by merging and screening of the detected frequency lines in each sub-lofargram based image stitching theory [17] and statistical model method [2]. The tracking results of simulation data and sea trial data prove that the proposed method can effectively reduce the amount of calculation and enhance the adaptability to time-varying signals while ensuring the performance under low SNR conditions.

## 2. Frequency Line Detection Using HMM

### 2.1. Elements of Hidden Markov Model

A Hidden Markov Model (HMM) is a probabilistic model for sequential data with an underlying hidden structure [18]. HMM can usually be characterized by the following five-dimensional set: {Q,V,A,B,Π}, $Q=\left\{q_{1},q_{2},\;\ldots ,\;q_{N}\right\}$ is a collection of all finite states, where N is the number of states; $V=\left\{v_{1},\;v_{2},\;\ldots ,\;v_{M}\right\}$ is the finite measurement set, where M is the total number of measurements; $A={\left[a_{ij}\right]}_{N\times N}$ is its transition probability matrix, where $a_{ij}=P_{r}\left(q_{j}\;at\;t+1|q_{i}\;at\;t\right)$ means the probability that the chain transitions from state $q_{i}$ at time t to State $q_{j}$ at time t+1. Note that the transition probabilities $a_{ij}$ are independent of time t ; $B={\left[b_{j}\left(k\right)\right]}_{N\times M}$ denote the measurement probability matrix, where $b_{j}\left(k\right)=P_{r}\left(v_{k}\;at\;t|q_{j}\;at\;t\right)\;$; $\Pi ={\left[\pi \left(i\right)\right]}_{N}$ is the initial state probability vector of the Markov chain. Simulation of an HMM measurement sequence of length T, given Π, A, and B, is straight forward. $I=\left\{i_{1},\;i_{2},\;\ldots ,\;i_{T}\right\},\;i_{t}\in Q$ denotes an arbitrary Markov chain state sequence of length T. $O=\left\{o_{1},o_{2},\ldots ,o_{T}\right\},\;o_{t}\in V$ is the measurement sequence, which is the only output from an HMM simulator. One of the main tasks of this paper is to estimate the sequence of frequency line states.

In the HMM theory, under the condition of the given observation state sequence O and the corresponding HMM parameters {A, B, Π}, the hidden state sequence I can be efficiently estimated by using the Viterbi algorithm [18] or the FB algorithm [19]. The former finds the optimal sequence for all state sequences, and the estimation result is $\hat{I}=arg\underset{I}{max}\{P(I|O)\},$ which has the meaning of the global optimal solution; The latter finds the maximum likelihood state ${\hat{i}}_{t},$ ${\hat{i}}_{t}=arg\underset{n\in N}{max}\{P(i_{t}=q_{n}|O)\}$ at each moment by forward-backward joint recursion, and then combines the estimated value ${\hat{i}}_{t}$ of each moment into a sequence of state estimation results $I=\left\{{\hat{i}}_{1},\;{\hat{i}}_{2},\;\ldots ,\;{\hat{i}}_{T}\right\},$ which has the meaning of a local optimal solution. When using HMM for frequency line tracking, the whole frequency range is divided into N frequency cells, the i -th frequency cell is represented as $\left[f_{i},f_{i+1}\right],\;i=1,\;\ldots ,\;N,$ and its center frequency is recorded as ${\tilde{f}}_{i}=\frac{f_{i}+f_{i+1}}{2}.$ When the spectral frequency f is located in this frequency interval, the state of the frequency line at this time is regarded as being in state i ; And the power spectrum data $z_{k}=\left(z_{k,0},\;z_{k,1},\;\ldots ,\;z_{k,N-1}\right)$ calculated at different times k is regarded as measurement information at different times, where $z_{k,i}=P\left(k,{\tilde{f}}_{i}\right).$ In the above formula, $P\left(k,{\tilde{f}}_{i}\right)$ represents the power spectral density corresponding to the frequency point ${\tilde{f}}_{i}$ at time k. Under the above conditions, the frequency line tracking problem in the lofargram is transformed into an estimation problem of the state in the HMM.

### 2.2. The Setting of HMM Parameters

In the calculation of the state transition probability matrix A, we assume that the state transition probability satisfies the Gaussian distribution $N\left(x;0,\sigma_{x}^{2}\right),$ where $\sigma_{x}^{2}$ represents the Gaussian process noise. According to the above assumption, the probability that the frequency line is shifted from the frequency state i to j is:

(1) $$g_{ij}=\frac{1}{\sqrt{2\pi }\sigma_{x}}\int_{f_{j}}^{f_{j+1}}e^{-\frac{{\left(f-{\tilde{f}}_{i}\right)}^{2}}{2\sigma_{x}^{2}}}df,\;i,j>0,\left|i-j\right|\leq R$$

where R refers to the maximum offset range of state i, and the probability of exceeding this range is uniformly set to zero. After normalization, the formula for calculating the elements in matrix A is obtained:

(2) $${\tilde{a}}_{ij}=\frac{g_{ij}}{\sum_{k=1}^{N}g_{ik}},\;i,j=1,\ldots ,N\;$$

As for the measurement probability matrix B, according to the literature [4], when the SNR is known,

(3) $$b_{i}\left(z_{k}\right)=\frac{I_{0}\left(\sqrt{\frac{4\rho_{k}Mz_{k,i}}{\sigma_{\epsilon }^{2}}}\right)}{\sum_{l=1}^{N}I_{0}\left(\sqrt{\frac{4\rho_{k}Mz_{k,l}}{\sigma_{\epsilon }^{2}}}\right)}$$

otherwise,

(4) $$b_{i}\left(z_{k}\right)=\frac{z\left(k,\;i\right)}{\sum_{j=1}^{N}z\left(k,\;j\right)}$$

In the above formula, $I_{0}$ represents a zero-order Bessel function.

In the traditional HMM-based frequency line detection process, since the a priori information of the frequency line parameters is usually lacking, the initial probability Π is set to be uniform, that is,

$$\pi \left(i\right)=\frac{1}{N}$$

After the above parameters settings, the frequency line can be tracked using the Viterbi algorithm or the FB algorithm. Although this processing method is suitable for the detection of single frequency line and the tracking of time-varying frequency line, there are problems such as large calculation amount and difficulty in detecting multiple frequency lines in global processing. Therefore, we have improved this method and proposed a set of new processing methods.

## 3. Improved Multiple Frequency Line Tracking Algorithm Based on HMM

**Figure 2:**
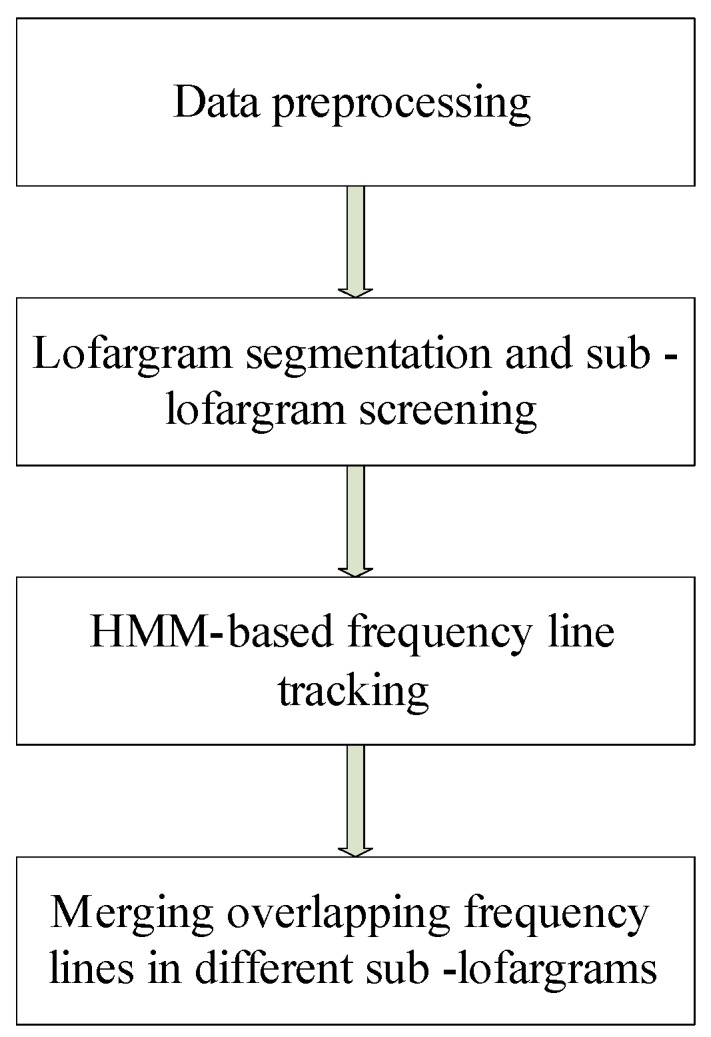
Improved multiple frequency line tracking algorithm based on hidden Markov model (HMM).

The algorithm consists of four steps, including (1) data preprocessing, (2) lofargram segmentation and sub-lofargram screening, (3) HMM-based frequency line tracking, and (4) merging overlapping frequency lines in different sub-lofargrams. The framework of the algorithm is shown in Figure 2.

In step 1, the lofargram is obtained by Short-time Fourier transform (STFT) on the received signal of passive sonar [20]. Then background equalization is performed to reduce the effect of background noise of marine environment. In step 2, the lofargram is segmented into several sub-lofargrams according to the preset sub-lofargram size and overlapping manner. Next, the number of frequency points in each sub-lofargram whose amplitude satisfies the threshold condition is counted and the HMM-based frequency line detection operation is performed only on the sub-lofargrams in which the number of frequency points is greater than the preset threshold. In step 3, the HMM-based viterbi algorithm is used to detect multiple frequency lines one by one in the sub-lofargrams screened out in the previous step, and then store the frequency line detection result of each sub-lofargram. In the last step, the frequency lines with overlapping parts detected by different sub-lofargrams are merged to obtain the final multiple frequency line detection results according to image stitching and statistical model method.

### 3.1. Data Preprocessing

The first step of data preprocessing is to perform STFT on the received signal using the method in [20] to obtain lofargram. The signal obtained by the passive sonar contains the target multiple frequency line components and the background noise of the marine environment. In order to accurately and reliably acquire multiple frequency lines, it is necessary to perform background equalization on the signal power spectrum set ${\left\{T_{k}\left(f\right)\right\}}_{k=1}^{K}$ in the lofargram to reduce the interference of noise on the line spectrum point tracking, where K is the number of power spectrums calculated in time series and $T_{k}\left(f\right)$ is the power spectrum at time *k*. In the case of background equalization, this paper first estimates the continuously changing noise background ${\left\{B_{k}\left(f\right)\right\}}_{k=1}^{K}.$ The estimation methods of noise background include post-sampling difference fitting, fitting method based on Empirical mode decomposition (EMD) [21] and fitting method based on regression parameters [22], etc. In this paper, the noise background estimation of the received signal is performed by the method described in [22]. Then subtract the background noise ${\left\{B_{k}\left(f\right)\right\}}_{k=1}^{K}$ in the power spectrum P(k,f) to obtain the difference spectrum ${\left\{P_{k}\left(f\right)\right\}}_{k=1}^{K}.$ The specific operation is

(5) $$P_{k}\left(f\right)=T_{k}\left(f\right)-B_{k}\left(f\right)$$

### 3.2. Lofargram Segmentation and Sub-Lofargram Screening

**Figure 3:**
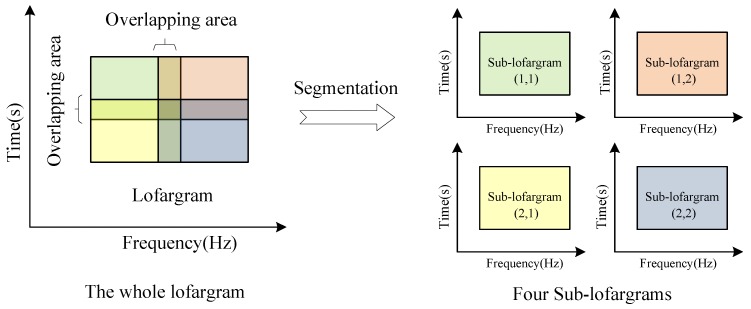
The segmentation of lofargram.

The process of lofargram segmentation is shown in Figure 3. The whole lofargram is segmented into several sub-lofargrams. There is a certain degree of overlap between adjacent sub-lofargrams in the horizontal direction (frequency direction) and the vertical direction (time direction). The size of the overlapping area of adjacent sub-lofargram is determined by actual needs. After processing, the whole lofargram is segmented into multiple small sub-lofargrams.

In order to reduce the amount of calculation during frequency line tracking, sub-lofargram screening is required before HMM-based frequency line tracking, and sub-lofargrams containing only noise signal will not be processed by further frequency line detection. The specific sub-lofargram screening operation is as follows.

**Figure 4:**
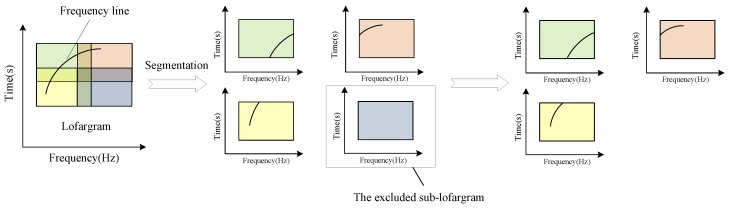
The segmentation of lofargram and sub-lofargram screening.

A high false alarm decision is made on the difference spectrum ${\left\{P_{k}\left(f\right)\right\}}_{k=1}^{K}$ using a preset threshold ${\left\{D_{k}\right\}}_{k=1}^{K}.$ Here $P_{k}\left(f\right)$ is the power difference spectrum at time k in each sub-lofargram. Then count the number of frequency points in each sub-lofargram that satisfy the decision condition. If the number is greater than a preset threshold $spotnum_{min},$ it is determined that there is a frequency line in the sub-lofargram, and the sub-lofargram is marked as a sub-lofargram to be processed; Otherwise, the sub-lofargram will be culled and no frequency line extraction will be performed on it. Figure 4 shows the flow of lofargram segmentation and sub-lofargram screening.

### 3.3. HMM-Based Frequency Line Tracking

After the lofargram is segmented and sub-lofargrams are screened, HMM-based frequency line tracking is then performed. In order to improve the efficiency of multi-frequency line detection, according to the difference of power spectrum amplitude between different frequency lines in each sub-lofargram, the Viterbi algorithm is used to sequentially extract a single frequency line until all existing frequency lines are extracted and stored.

**Figure 5:**
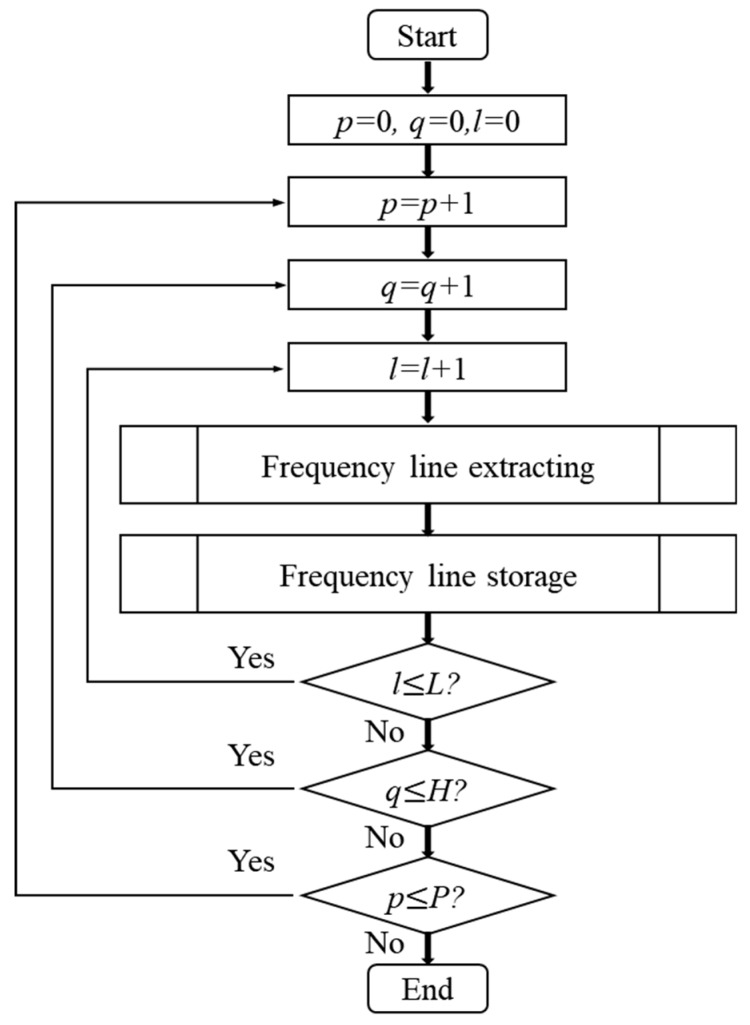
Frequency line tracking.

Before performing frequency line extraction, it is necessary to preset the maximum number of frequency lines L in the sub-lofargram to be detected according to the prior information. When performing frequency line tracking, it is assumed that there is only one frequency line in the sub-lofargram. Only one most likely frequency line is taken from the sub-lofargram at this time as the current frequency line tracking result. When tracking the next frequency line, the detected frequency line needs to be deleted from the sub-lofargram, and then the frequency lines is tracked in the generated new sub-lofargram until all possible L frequency lines are detected. The HMM-based frequency line tracking process is shown in Figure 5.

In the figure, p,q represents the sub-lofargram number, p=1, 2, …, P means the pth sub-lofargram in time direction, q=1, 2, …, H means the qth sub-lofargram in frequency direction. l=1, 2, …, L is the frequency line number in each sub-lofargram.

The processing method of each step will be described in detail below.

#### 3.3.1. Frequency Line Extracting

Using the Viterbi algorithm to perform frequency line extracting on the processed sub-lofargram first needs to initialize the initial state distribution probability of the sub-lofargram. Since the temporally adjacent sub-lofargrams are related to each other, the processing result of the previous sub-lofargrams can be used as a priori information to set the initial probability of the unprocessed sub-lofargrams. If the sub-lofargrams to be processed has adjacent processed sub-lofargrams in the vertical direction (time direction), $\pi \left(i\right)=P(x_{K}^{p,q-1}=i|z_{1}^{p,q-1},\;\ldots ,\;z_{K}^{p,q-1}),$ otherwise $\pi \left(i\right)=\frac{1}{N}.$

For sub-lofargram with number (p,q), in order to obtain the most likely sequence of states, first import two variables δ and θ. Defining the maximum probability of all single paths with state i at time k is

(6) $$\delta_{k}\left(i\right)=\delta_{k}\left(x_{k}^{p,q}=i\right)=maxP\left(x_{k}^{p,q}=i,x_{k-1}^{p,q},\ldots ,x_{1}^{p,q},z_{k}^{p,q},\ldots ,z_{1}^{p,q}|\lambda \right)i=1,\;2,\ldots ,N$$

In order to generate the final state sequence, the (k−1)th node defining the path with the highest probability among all the single paths with the state i at time k is

(7) $$\theta_{k}\left(i\right)=argmax\left[\delta_{k-1}\left(j\right)a_{ji}\right],\;i=1,\;2,\ldots ,N\;$$

Next, the optimal frequency line trajectory is obtained by iterating through the Viterbi algorithm as follows.

1. Calculate the HMM parameters {A,B,Π} according to the parameter setting method described in the first section. Initialize the variables δ and θ corresponding to all states in the state space.
  (8) $$\delta_{1}\left(i\right)=\pi \left(i\right)P\left(z_{1}^{p,q}|x_{1}^{p,q}=i\right)=\pi \left(i\right)b_{i}\left(z_{1}^{p,q}\right),\;i=1,\;2,\ldots ,N\;$$
  (9) $$\;\theta_{1}\left(i\right)=0,\;i=1,\;2,\ldots ,N$$
2. Recursive backward, for each state j at time k=2, 3,⋯,K, $\delta_{k}\left(j\right)$ is calculated and its previous state is stored.
  (10) $$\delta_{k}\left(j\right)=\underset{1\leq i\leq N}{max}\left[\delta_{k-1}\left(i\right)a_{ij}\right]P\left(z_{k}^{p,q}|x_{k}^{p,q}=j\right)=\underset{1\leq i\leq N}{max}\left[\delta_{k-1}\left(i\right)a_{ij}\right]b_{j}\left(z_{k}^{p,q}\right),\;j=1,2,\ldots ,N\;\left(10\right)$$
  (11) $$\;\theta_{k}\left(j\right)=\underset{1\leq i\leq N}{argmax}\left[\delta_{k-1}\left(i\right)a_{ij}\right],\;j=1,\;2,\ldots ,N\;\;$$
3. After the recursion is completed, the state estimate at time K is found by maximizing $\delta_{K}\left(j\right),$ and the path of the final state is traced back to the initial time by the backward pointer stored in $\theta_{K}.$
  (12) $$P^{*}=\underset{1\leq j\leq N}{max}\delta_{K}\left(x_{K}^{p,q}=j\right)$$
  (13) $$\;{\hat{x}}_{K}^{p,q}=\underset{1\leq j\leq N}{argmax}\left[\delta_{K}\left(j\right)\right]\;$$
4. Backtracking to get the optimal path. For k=K−1,K−2,…,1,
  (14) $${\hat{x}}_{k}^{p,q}=\theta_{k+1}\left({\hat{x}}_{k+1}^{p,q}\right)\left(14\right)$$
  The current detection frequency line of the sub-lofargram with number (p,q) is
  $$X^{p,q}=\left({\hat{x}}_{1}^{p,q},{\hat{x}}_{2}^{p,q},\ldots ,{\hat{x}}_{K}^{p,q}\right)$$
5. The amplitude-based decision method is used to determine the start, end time and trajectory rationality of the detected trajectory.

According to the Viterbi algorithm, an optimal frequency line from time frame 1 to time frame K can be obtained after step 1~4. However, the frequency points on the detected frequency lines are not necessarily generated by the actual narrowband signals. Therefore, it is necessary to make a second decision point by point on all frequency points on the frequency line. Assuming that the frequency of the point to be determined is ${\hat{f}}_{m},$ the neighborhood of the M point is expressed as $\left[{\hat{f}}_{m}-M,\;{\hat{f}}_{m}+M\right],$ and the power spectral density corresponding to the frequency point in the neighborhood is denoted as $P_{l}$. According to the local 3σ criterion, the frequency line point rationality and the trajectory start and end time are determined as follows.

(15) $$P_{k}\left({\hat{f}}_{m}\right)=\{\begin{array}{c} \mathrm{Frequency}\;\mathrm{line}\;\mathrm{point},\;\;\;P_{k}\left({\hat{f}}_{m}\right)\geq \bar{P_{l}}+3\sigma_{S}\\ \mathrm{False}\;\mathrm{alarm},\;\;\;P_{k}\left({\hat{f}}_{m}\right)\leq \bar{P_{l}}+3\sigma_{S} \end{array}$$

where $\bar{P_{l}}$ is the average of the power spectral density corresponding to the 2M frequency points contained in the ${\hat{f}}_{m}$ neighborhood $\left[{\hat{f}}_{m}-M,\;{\hat{f}}_{m}+M\right].$

#### 3.3.2. Frequency Line Storage

According to the position of the frequency line in the sub-lofargram, the frequency point that satisfies the condition is stored in the set $W^{p,q}=\left\{W_{l}^{p,q}\right\},$ where l=1,…L ; $W_{l}^{p,q}=\left\{W_{total}^{p,q},W_{local}^{p,q},W_{right}^{p,q},W_{down}^{p,q}\right\}.$ The storage of line points in $W_{l}^{p,q}$ is related to the way the sub-lofargrams overlap. When the horizontal overlapping length is $N_{H}$ frequency points and the vertical direction overlaps $K_{V}$ time frames, the components of $W_{l}^{p,q}$ are defined as follows.

$W_{local}^{p,q}$ stores the frequency points in the middle of the sub-lofargram among these frequency points,

(16) $$W_{local}^{p,q}=\left\{\left[k,spot_{p,q}^{l}\left(k\right)\right],s.t.\;spot_{p,q}^{l}\left(k\right)={\hat{x}}_{k}^{p,q}\right\}$$

In the above formula,

$${\hat{x}}_{k}^{p,q}\in X^{p,q},N_{H}/2<{\hat{x}}_{k}^{p,q}\leq N-N_{H}/2\&K_{V}/2<k\leq K-K_{V}/2$$

$\;W_{down}^{p,q}$ stores the frequency points on the underside of the sub-lofargram among these frequency points,

(17) $$W_{right}^{p,q}=\left\{\left[k,spot_{p,q}^{l}\left(k\right)\right],s.t.\;spot_{p,q}^{l}\left(k\right)={\hat{x}}_{k}^{p,q}\right\}$$

In the above formula,

$${\hat{x}}_{k}^{p,q}\in X^{p,q},\;{\hat{x}}_{k}^{p,q}>N-N_{H}/2\&K_{V}/2<k\leq K-K_{V}/2$$

$\;W_{down}^{p,q}$ stores the frequency points on the underside of the sub-lofargram among these frequency points,

(18) $$W_{down}^{p,q}=\left\{\left[k,spot_{p,q}^{l}\left(k\right)\right],s.t.\;spot_{p,q}^{l}\left(k\right)={\hat{x}}_{k}^{p,q}\right\}$$

In the above formula,

$${\hat{x}}_{k}^{p,q}\in X^{p,q},N_{H}/2<{\hat{x}}_{k}^{p,q}\leq N-N_{H}/2\&k>K-K_{V}/2$$

$\;W_{total}^{p,q}$ stores all the frequency points in the trajectory detection that meet the conditions,

(19) $$W_{total}^{p,q}=\left\{W_{local}^{p,q},W_{right}^{p,q},W_{down}^{p,q}\right\}$$

In $\left[k,spot_{p,q}^{l}\left(k\right)\right],$ k is the time frame number of the trajectory line point; $spot_{p,q}^{l}\left(k\right)$ represents the detected frequency position of the lth frequency line of the sub-lofargram whose position number is (p,q) when the time is k. That is to say, $spot_{p,q}^{l}\left(k\right)$ is numerically equal to ${\hat{x}}_{k}^{p,q}.$

**Figure 6:**
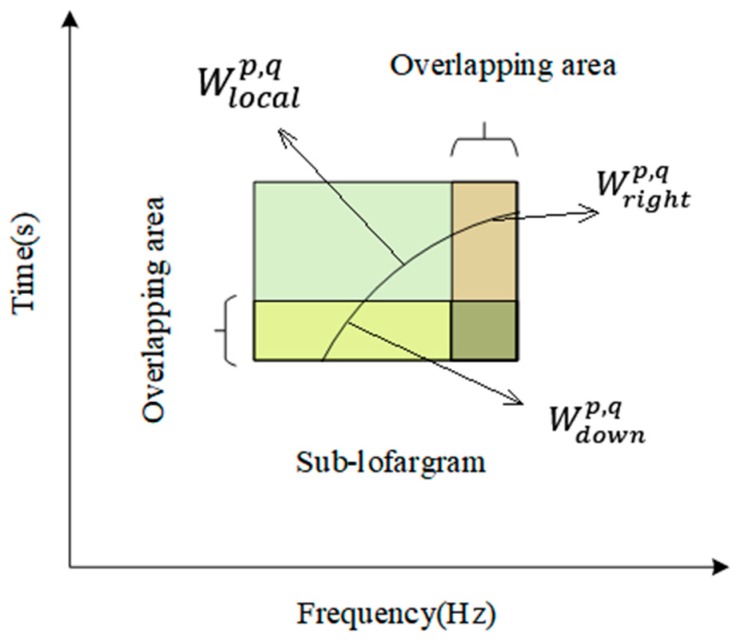
Diagram of different frequency point sets in sub-lofargram.

The position of the above-mentioned frequency point set in a sub-lofargram is as shown in Figure 6.

### 3.4. Merging Overlapping Frequency Lines in Different Sub-Lofargrams

Finally, the frequency lines detected by all sub-lofargrams are combined by image stitching [17] and statistical model method [2]. Since there is a certain degree of overlap in the frequency domain direction and the time domain direction when the lofargram is segmented, the $W_{total}^{p,q}$ in the frequency point set $W_{l}^{p,q}$ of all the sub-lofargrams is merged together to form a complete frequency line detection result.

The main difficulty in frequency line merging is to determine the correspondence between different frequency lines of adjacent sub-lofargrams. In this paper, each sub-lofargrams is treated as a sub-image, and the final frequency line merge result is the result of image mosaic of different sub-images.

**Figure 7:**
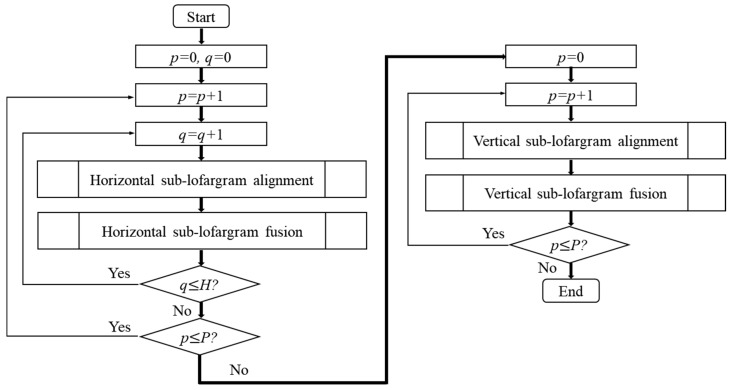
Merging overlapping frequency lines in different sub-lofargram.

The process of merging overlapping frequency lines in different sub-lofargram is shown in Figure 7.

It can be seen from the above figure that in the process of merging overlapping frequency lines, the overlapping frequency lines in adjacent sub-lofargrams in the horizontal direction (frequency direction) are first merged. Then we merge the overlapping frequency lines in adjacent sub-lofargrams in the vertical direction (time direction). The specific operations of sub-lofargram alignment and sub-lofargram fusion are described in detail below.

#### 3.4.1. Sub-Lofargram Alignment

In the image alignment process, image alignment is performed on adjacent sub-lofargram by using the spatial domain-based template matching method. In the process of registration of adjacent sub-lofargrams, it is necessary to extract feature points of overlapping portions of adjacent images and form feature point pairs. Here, the frequency point of the overlapping portion of adjacent sub-lofargrams detected previously is taken as the feature point. The distance matrix between the different frequency lines of the adjacent sub-lofargrams is calculated to perform image feature point matching, that is, frequency line matching. The definition of the frequency line distance matrix is as follows:

(20) $$J_{i-1,i}=\left[\begin{array}{cccc} \Delta_{11} & \Delta_{12} & \cdots & \;\Delta_{1L_{i}}\\ \Delta_{21} & \Delta_{22} & \cdots & \Delta_{2L_{i}}\\ \vdots & \vdots & \ddots & \vdots \\ \Delta_{L_{i-1}1} & \Delta_{L_{i-1}2} & \cdots & \Delta_{L_{i-1}L_{i}} \end{array}\right]$$

where $L_{i}$ represents the number of frequency lines detected by the i -th sub-lofargram, and $\Delta_{j_{i-1}r_{i}}$ represents the distance between the j -th frequency line of the (i−1) -th sub-lofargram and the r -th frequency line of the i -th sub-lofargram. The calculation method of $\Delta_{j_{i-1}r_{i}}$ is related to the way the frequency lines is merged.

When the frequency lines are horizontally merged, that is, combined in the frequency direction, the two sub-lofargrams to be feature point matching are left and right adjacent in the whole lofargram.

The calculation of $\Delta_{j_{i-1}r_{i}}$ is related to $W_{local}^{p,q}$ of the r -th frequency line of the (i−1) -th sub-lofargram and $W_{right}^{p,q-1}$ of the j -th frequency line of the i -th sub-lofargram. Its value is the average of the frequency differences of frequency points of the same time frames in the two frequency point sets.

(21) $$\Delta_{j_{i-1}r_{i}}=\frac{\sum_{s=1}^{spotnum}\left|spot_{i-1}^{j}\left(k_{s}\right)-spot_{i}^{r}\left(k_{s}\right)\right|}{spotnum}$$

(22) $$\;\left[k_{s},spot_{i-1}^{j}\left(k_{s}\right)\right]\in W_{down}^{p-1,q};W_{down}^{p-1,q}\in W_{j}^{p-1,q};\left[k_{s},spot_{i}^{r}\left(k_{s}\right)\right]\in W_{local}^{p,q};W_{local}^{p,q}\in W_{r}^{p,q}\;$$

In the above formula, spotnum is the number of frequency points with the same time frame among the two frequency lines; $spot_{i}^{r}\left(k_{s}\right)$ is the frequency position of the s -th frequency of the r -th track of the i -th sub-lofargram with the same time frame. The range of s is 1≤s≤spotnum. $k_{s}$ is the time frame number of the frequency point on the frequency line.

After the frequency line distance matrix is calculated, the sub-lofargram feature points can be matched by using the elements in the matrix, that is, the frequency lines in adjacent sub-lofargrams are matched. The matching process is as follows:

Extract the j -th line of the distance matrix $J_{i-1,i}$ and record it as

(23) $$V_{j}=J_{i-1,i}\left(j,:\right)=\left(\Delta_{j1},\Delta_{j2},\Delta_{j3},\ldots ,\Delta_{jL_{i}}\right),\;1\leq j\leq L_{i-1}$$

Find the minimum value in $V_{j}$ and determine if this value is still the minimum value in the corresponding column of the frequency line distance matrix. If this condition is met, the *j*-th frequency line in (i−1) -th sub-lofargram and the r -th frequency line in i -th sub-lofargram are the best matching frequency lines in the two sub-lofargrams. If the minimum value is less than the preset frequency fluctuation threshold, it indicates that the two frequency lines are successfully matched and can form a feature point pair. After the feature point pair is successfully matched, all elements of the j -th row corresponding to the frequency line distance matrix and all elements of the r -th column are set to a large value. Then iteratively recalculates the next pair of feature points until all matching frequency lines are found.

#### 3.4.2. Sub-Lofargram Fusion

**Figure 8:**
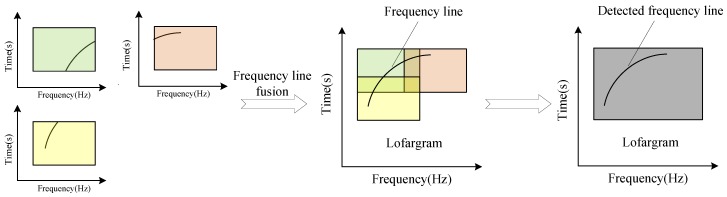
Frequency line fusion between adjacent sub-lofargrams.

After the matching of the feature points of the adjacent sub-lofargrams, the image alignment work is completed. Next, sub-lofargram fusion is performed, mainly to fuse the frequency point set $W^{p,q}$ of two adjacent sub-lofargrams. Figure 8 shows the process of frequency line fusion.

In the process of horizontal sub-lofargram fusion, that is, when $W^{p,q-1}$ and $W^{p,q}$ are merged, the successfully matched frequency lines $W_{j}^{p,q-1}$ and $W_{r}^{p,q}$ are first merged.

When merging matched $W_{j}^{p,q-1}$ and $W_{r}^{p,q},$ the $W_{total},W_{local},\;W_{down}$ parts of the two frequency lines are merged by the direct averaging method. After the combination, the $W_{j}$ corresponding position line spectral point data is re-assigned and stored. That is

(24) $$\begin{array}{l} \left[k,{\tilde{spot}}_{i-1}^{j}\left(k\right)\right]\\ =\{\begin{array}{c} \left[k,spot_{i-1}^{j}\left(k\right)\right],\;\&spot_{i-1}^{j}\left(k\right)\in W_{symbol}^{p,q-1}\\ \left[k,\left[spot_{i-1}^{j}\left(k\right)+spot_{i}^{r}\left(k\right)\right]/2\right],\;spot_{i-1}^{j}\left(k\right),spot_{i}^{r}\left(k\right)\in W_{symbol}^{p,q-1}\cap W_{symbol}^{p,q}\\ \left[k,spot_{i}^{r}\left(k\right)\right],\;\&spot_{i}^{r}\left(s,k\right)\in W_{symbol}^{p,q} \end{array} \end{array}$$

(25) $$\;W_{symbol}^{p,q-1}=\left\{\left[k,{\tilde{spot}}_{i-1}^{j}\left(k\right)\right]\right\}\;$$

where, symbol∈{total,local,down}.

Then replace the $W_{right}^{p,q-1}$ part of $W_{j}^{p,q-1}$ with $W_{right}^{p,q}$ of $W_{r}^{p,q}.$

Finally, the unmatched frequency points in $W^{p,q}$ are added to $W^{p,q-1},$ and the fusion of the matching frequency lines is completed. That is

(26) $$W_{L_{p,q-1}+lnum}^{p,q-1}=W_{lnum}^{p,q},1\leq lnum\leq Lnum$$

where, lnum is the unmatched frequency line number in sub-lofargram (*p*,*q*), and Lnum is the number of unmatched frequency lines. After updating $L_{p,q-1}$ to $L_{p,q-1}+Lnum,$ the resulting $W^{p,q-1}=\left\{W_{l_{p,q-1}}^{p,q-1}\right\},1\leq l_{p,q-1}\leq L_{p,q-1}$ is the result of combining the frequency line sets. $PHW^{p,q}$ obtain P horizontally merged track sets $W^{1}=\left\{W^{p,1}\right\},\;0<p\leq P$ after the horizontal track sub-image stitching is completed.

The alignment and fusion of sub-lofargram in the vertical direction is basically the same as the process in horizontal direction.

The difference is that in the frequency line matching process, the frequency line distance matrix is generated by using the $W_{local}^{p,1}$ part of $W_{r}^{p,1}$ and the $W_{down}^{p-1,1}$ part of $W_{j}^{p-1,1}\;$; When the frequency lines are combined, the $W_{total},\;W_{local},\;W_{right}$ portions of the two frequency line sets are matched and stored in the corresponding position of $W_{j}^{p-1,1}.$ Then, the $W_{down}^{p-1,1}$ part of $W_{j}^{p-1,1}$ is replaced by $W_{down}^{p,1}$ of $W_{r}^{p,1}$ to complete the fusion of the vertical matching frequency lines. Finally, the unmatched frequency lines in $W^{p,1}$ are added to $W^{p-1,1},$ and $W^{p-1,1}$ is the result of the frequency line merging of adjacent sub-lofargram in horizontal direction. The $W^{1,1}$ obtained after iterative merging is the final result of frequency line merging.

Then, the final frequency line merging result is judged again to eliminate the fusion frequency line whose frequency line length does not satisfy the condition. The retained track is the result of tracking multiple frequency lines.

## 4. Performance Evaluation

### 4.1. Calculation Analysis

In the above frequency line detection process, the main calculation amount is distributed in the process of extracting the frequency line by using the Viterbi algorithm. If the conventional Viterbi algorithm is used for extraction one frequency line in an lofargram of N×K dimension, the total number of multiplications is $\left(5K-4\right)N^{2}+\left(K+1\right)N,$ the total addition numberis $\left(3K-2\right)N^{2}+\left(2-3K\right)N,$ and the total number of assignments is 3KN. The N×K dimension indicates that there are K time frames in the time direction of lofargram and N frequency points in the frequency direction of lofargram.

(1) The following is a calculation amount analysis when extracting multiple frequency lines in a lofargram with N×K dimension by using the method proposed in this paper. Assume that the number of preset frequency lines in the lofargram is L.
  Let each sub-lofargram contain $K_{M}$ time frames and $N_{D}$ frequency points. The overlap mode is set to overlap 1/2 sub-lofargram in both the time direction and the frequency direction. At this time, the number of sub-lofargrams divided in the frequency direction is $D=2N/N_{D}-1\;$; the number of sub-lofargrams divided in the time direction is $M=2K/K_{M}-1.$ The preset number of frequency lines for frequency line extracting in a sub-lofargram grid is $LN_{D}/N.$
  When performing frequency line extraction operation in one sub-lofargram, the number of multiplications is $\frac{LN_{D}}{N}\left(\left(5K_{M}-4\right)N_{D}{}^{2}+\left(K_{M}+1\right)N_{D}\right),$ the number of additions is $\frac{LN_{D}}{N}\left(\left(3K_{M}-2\right)N_{D}{}^{2}+\left(2-3K_{M}\right)N_{D}\right)$ and the number of assignments is $\frac{LN_{D}}{N}\left(3K_{M}N_{D}\right).$ Since there are a total of D×M sub-lofargrams, when using the method proposed in this paper for frequency line extracting in sub-lofargrams, the total number of multiplications is
  (27) $$Cal_{Mul}=\frac{\left(2N-N_{D}\right)\left(2K-K_{M}\right)L}{NK_{M}}\left[\left(5K_{M}-4\right)N_{D}{}^{2}+\left(K_{M}+1\right)N_{D}\right]$$
  the total number of additions is
  (28) $$Cal_{Add}=\frac{\left(2N-N_{D}\right)\left(2K-K_{M}\right)L}{NK_{M}}\left[\left(3K_{M}-2\right)N_{D}{}^{2}+\left(2-3K_{M}\right)N_{D}\right]$$
  the total number of assignments is
  (29) $$Cal_{Assign}=\frac{\left(2N-N_{D}\right)\left(2K-K_{M}\right)L}{N}\left(3N_{D}\right)$$
  According to the above formula, when $K_{M}\ll K$ and $N_{D}\ll \;N,$ the algorithm complexity in frequency line tracking is $o\left(KLN_{D}{}^{2}\right).$ The computational complexity in merging overlapping frequency lines in different sub-lofargrams is mainly concentrated in the calculation of the distance divergence matrix, and the algorithm complexity of this part is $o\left(L^{2}\right).$ Therefore, the algorithm complexity of the proposed method is about $o\left(KLN_{D}{}^{2}+L^{2}\right).$
(2) If the traditional HMM-based method is used to extract the frequency lines in lofargram under the same conditions. As L frequency lines are to be extracted in the entire lofargram, the total number of multiplications is
  (30) $$Caltr_{Mul}=L\left[\left(5K-4\right)N^{2}+\left(K+1\right)N\right]$$
  the total number of additions is
  (31) $$Caltr_{Add}=L\left[\left(3K-2\right)N^{2}+\left(2-3K\right)N\right]$$
  the total number of assignments is
  (32) $$Caltr_{Assign}=3LKN$$
  Therefore, the algorithm complexity of the traditional method in frequency line tracking is about $o\left(KLN^{2}\right).$

According to the analysis of (1) and (2), the algorithm complexity of the proposed method is about $o\left(KLN_{D}{}^{2}+L^{2}\right)$ and the algorithm complexity of the traditional method in frequency line tracking is about $o\left(KLN^{2}\right).$ Since L≪N and $N_{D}$ are in multiple relationship with N, The method proposed in this paper obviously has lower algorithm complexity.

Replace $N_{D}$ and $K_{M}$ in the case of (1) with 2N/D+1 and 2K/M+1, and the formula (27) to (29) is converted to

(33) $$Cal_{Mul}=16L\frac{DM}{\left(D+1\right)\left(M+1\right)}\left[\left(5K-2\left(M+1\right)\right)\frac{N^{2}}{{\left(D+1\right)}^{2}}+\left(K+\frac{M+1}{2}\right)\frac{N}{2}\right]$$

(34) $$\;Cal_{Add}=16L\frac{DM}{\left(D+1\right)\left(M+1\right)}\left[\left(3K-2\left(M+1\right)\right)\frac{N^{2}}{{\left(D+1\right)}^{2}}+\left(M+1-3K\right)\frac{N}{2}\right]\;$$

(35) $$\;Cal_{Assign}=\frac{8DM*3LKN}{{\left(D+1\right)}^{2}\left(M+1\right)}\;$$

When M≫1, D≫1 and M≪K, $\frac{DM}{\left(D+1\right)\left(M+1\right)}\approx 1.$ In this case, the approximate calculation amount of the frequency line extraction process using two algorithms is as shown in Table 1.

As can be seen from the above table, under ideal conditions, the calculation amount of the traditional HMM-based method is about $D^{2}/16$ times that of the method proposed in this paper.

However, in the whole frequency line tracking process, the proposed method also includes the processes of sub-lofargram alignment and sub-lofargram fusion etc. These steps also increase the amount of calculation when the algorithm is executed. Next, the actual execution time of the two different algorithms was measured on the MATLAB simulation platform.

During the simulation, a set of simulation signals with a sampling rate of 10 kHz are generated, and the background noise is colored noise. The lofargram to be detected contains a total number of time frames of 100 (time span of 80 s), a total frequency state number of 2816 (frequency span of 1719 Hz), and a preset number of existing frequency lines of 20. When using the method of this paper, each sub-lofargram is 40 × 512 dimensions, that is, there are 40 time frames in the time direction (time span is 32 s), and there are 512 frequency points in the frequency direction (frequency span is 312 Hz). The overlap time frame length between adjacent sub-lofargram is 20, and the overlap frequency point length is 256. Under this condition, the total execution time of the algorithm is approximately 11.4 s. The time taken to perform frequency line tracking using the traditional HMM-based method is about 44.0 s. According to the above time-consuming comparison, we can conclude that the frequency line tracking method proposed in this paper is much more computationally efficient than the traditional HMM-based method.

### 4.2. Detection Performance Comparison

A set of simulated signals is generated whose background noise is colored noise. The sampling rate of the signal is 10 kHz. The number of frequency line signals included in the simulation signal is L = 4. The frequency lines near the frequency of 300 Hz are continuous frequency lines with fluctuations, and the frequency lines around the frequencies of 100 Hz, 200 Hz, and 400 Hz are pulsed time-varying frequency lines. Each sub-lofargram has a size of 40 time frames (time span of 32 s) and 512 frequency points (frequency span of 312 Hz). The overlap time frame length between adjacent sub-lofargram is 20, and the overlap frequency point length is 256. The SNR after background equalization is taken as −20 dB.

**Figure 9:**
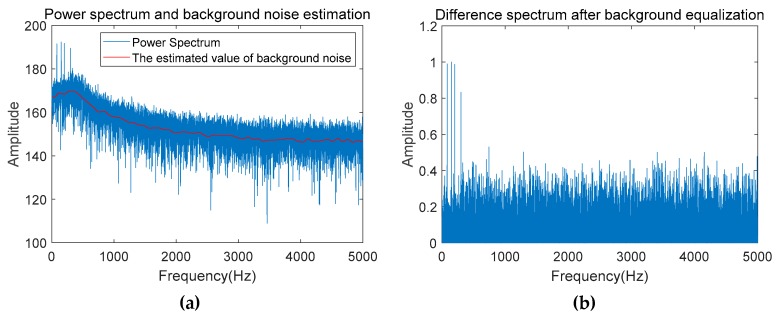
Comparison of power spectrum data: (**a**) Before background equalization; (**b**) After background equalization.

Figure 9 shows the original power spectrum of a certain frame of data and the difference spectrum obtained after background equalization.

**Figure 10:**
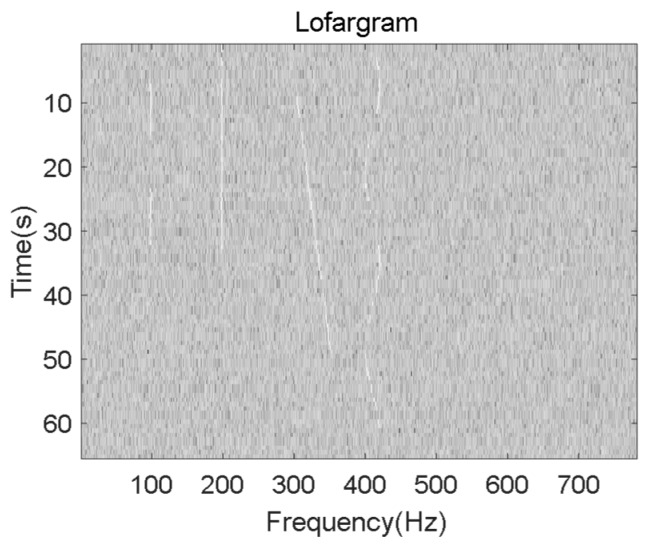
Lofargram after background equalization.

Since the simulated frequency line signals only exist in the low frequency part, in order to display the frequency line extraction result more intuitively, the analysis of the lofargram is mainly concentrated in the rectangular lofargram with the frequency range of 0~782 Hz and the time range of 0~65 s. The lofargram obtained after background equalization is shown in Figure 10.

**Figure 11:**
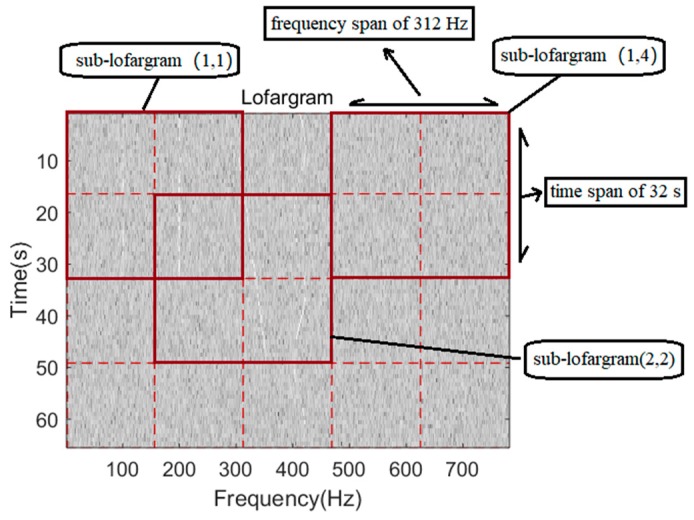
Segmentation.

The lofargram is segmented as shown in Figure 11.

**Figure 12:**
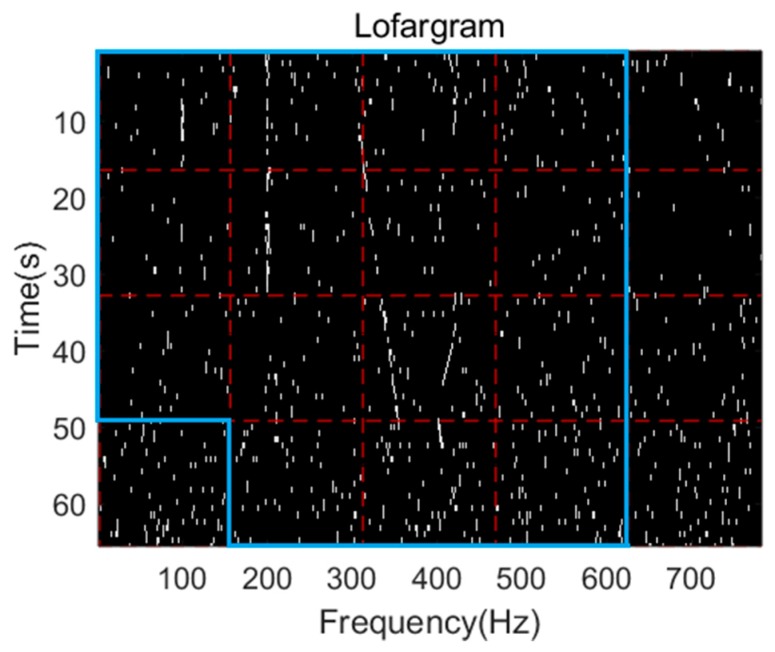
Lofargram and reserved sub-lofargram after screening.

A high false alarm decision is made for each frequency point in the lofargram above, and the binarized lofargram obtained after the decision and the screening result are shown in Figure 12.

In the above figure, each white point indicates the frequency point that satisfies the initial detection condition. The number of white points in each sub-lofargram in the graph is then compared to a preset threshold to screen out the sub-lofargram for which frequency line detection is required. The area selected by the blue wire is the area where the selected sub-lofargrams exist.

**Figure 13:**
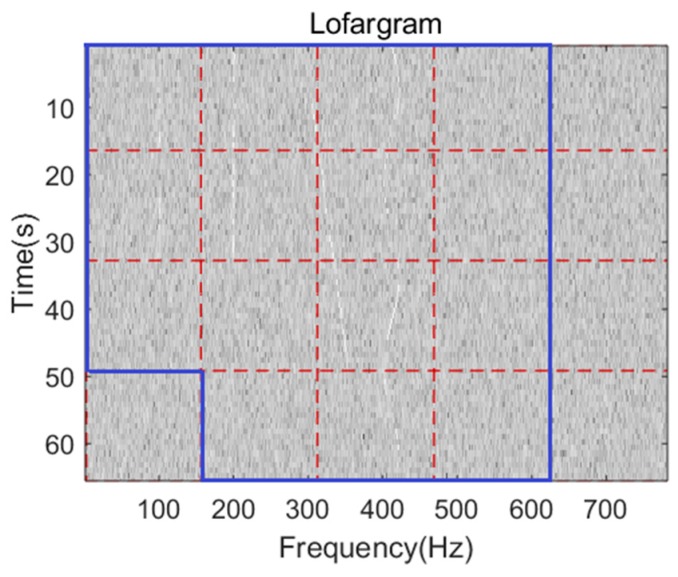
Lofargram screening results.

In the original lofargram, the location of the sub-lofargram that requires frequency tracking is shown in Figure 13.

In the above figure, the blue line selection area is the area of the sub-lofargram to be processed in the original lofargram. When the frequency line in the sub-lofargram is tracked using the Viterbi algorithm, the model parameters are set as described in Section 2.2. The elements in the state transition matrix A satisfy a Gaussian distribution with a mean of 0 and a variance of 1.

**Figure 14:**
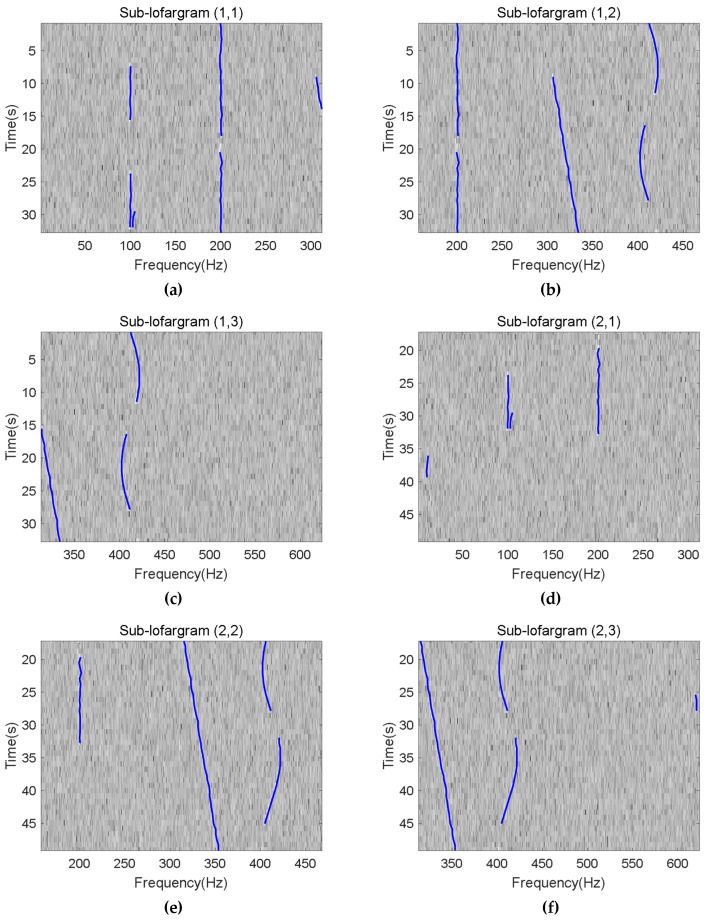
Line tracking results from sub-lofargram (1,1) to sub-lofargram (2,3): (**a**) Sub-lofargram (1,1); (**b**) Sub-lofargram (1,2); (**c**) Sub-lofargram (1,3); (**d**) Sub-lofargram (2,1); (**e**) Sub-lofargram (2,2); (**f**) Sub-lofargram (2,3).

Figure 14 shows the frequency line extraction results of six adjacent sub-lofargrams.

**Figure 15:**
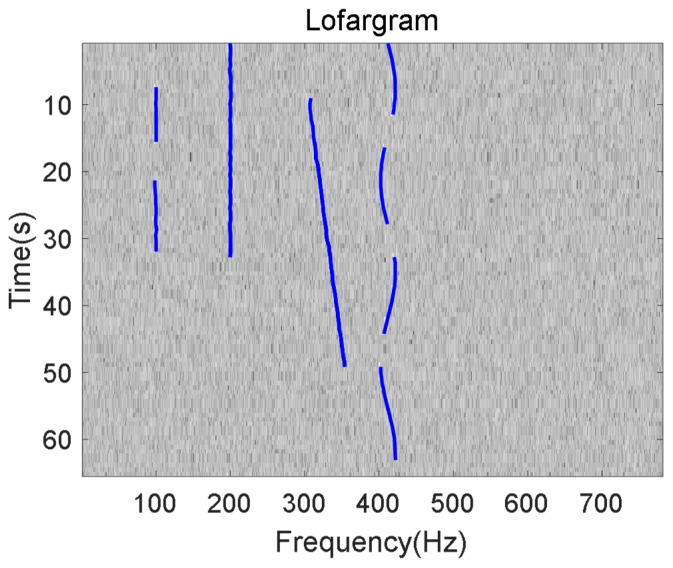
Final frequency line tracking results of proposed method.

Finally, the frequency line detection result of the rectangular simulated lofargram with the frequency range of 0~782 Hz and the time range of 0~65 s is shown in Figure 15.

**Figure 16:**
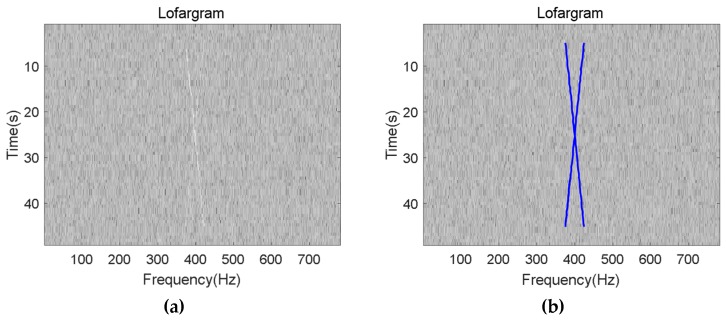
Line detection result of different method: (**a**) Traditional HMM-based method; (**b**) The method proposed in this paper.

The blue curve in the above figure is the detected frequency line. It can be seen that the method proposed in this paper has good detection performance for different types of frequency line signals. When the SNR after background equalization is −24dB, the comparison of the frequency line detection result between traditional HMM-based method and the method proposed in this paper is shown in Figure 16.

It can be seen from the above comparison figure that the proposed method can not only effectively reduce the amount of calculation, but also has better adaptability to the pulse signal that may exist.

**Figure 17:**
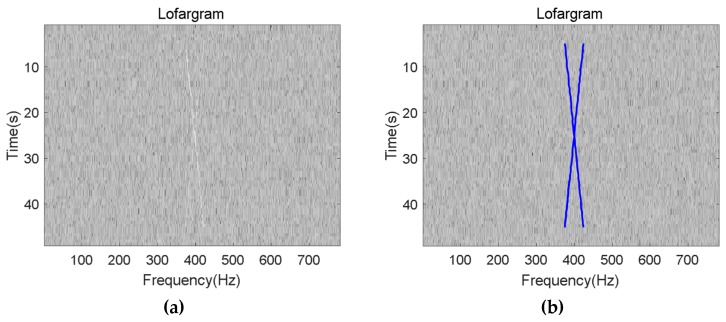
Detection result of the cross-signal: (**a**) Lofargram after background equalization; (**b**) The detection result.

Figure 17 shows the results of the detection of the cross-frequency line components contained in the signal using the proposed methods in this paper under a SNR of −20 dB after background equalization. The test results show that this method still has good detection performance for signals containing complex frequency line components.

In order to more intuitively compare the performance of different types of algorithms, define the probability of detection and the probability of false alarms as follows

(36) $$P_{D}=\frac{\mathrm{Number}\;\mathrm{of}\;\mathrm{detected}\;\mathrm{frequency}\;\mathrm{points}}{\mathrm{Number}\;\mathrm{of}\;\mathrm{real}\;\mathrm{frequency}\;\mathrm{points}}\times 100\%\;$$

(37) $$\;P_{F}=\frac{\mathrm{Number}\;\mathrm{of}\;\mathrm{error}\;\mathrm{detections}}{\mathrm{Total}\;\mathrm{number}\;\mathrm{of}\;\mathrm{detected}\;\mathrm{points}}\times 100\%$$

**Figure 18:**
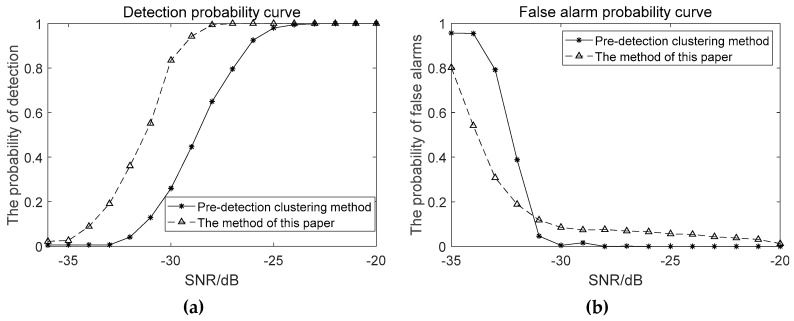
Performance comparison between the proposed method in this paper and pre-detection clustering method: (**a**) The detection probability curve; (**b**) The false alarm probability curve.

Algorithmic performance evaluation was performed using four frequency lines around 100, 200, 300, and 500 Hz in the simulated lofargram above. When the line-spectrum SNR is −36~−20dB, the performance curve corresponding to the proposed method in this paper and the conventional pre-detection clustering method [1,2] is shown in Figure 18.

As can be seen from the above performance comparison, the proposed method has better tracking frequency line capability under low SNR conditions than the conventional clustering method.

**Figure 19:**
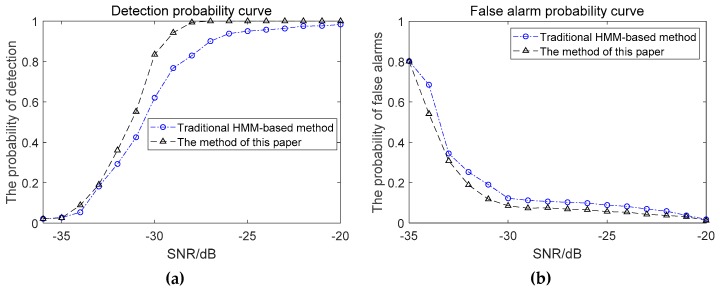
Performance Comparison of the proposed method in this paper and traditional HMM-based method between different method: (**a**) The detection probability curve; (**b**) The false alarm probability curve.

When the SNR is −36~−20dB, the detection probability curve and the false alarm probability curve of the proposed method and traditional HMM-based method are as shown in Figure 19.

It can be seen from Figure 19 that under the condition of weak time-varying signals, the false alarm probabilities of the two meshing methods are basically the same. The proposed method enhances the connection between adjacent sub-lofargrams, effectively increases the detection probability and improves the spectral line detection performance. However, the amount of calculation by this method has also increased. In the actual frequency line detection process, the amount of overlap needs to be determined according to the specific situation.

**Figure 20:**
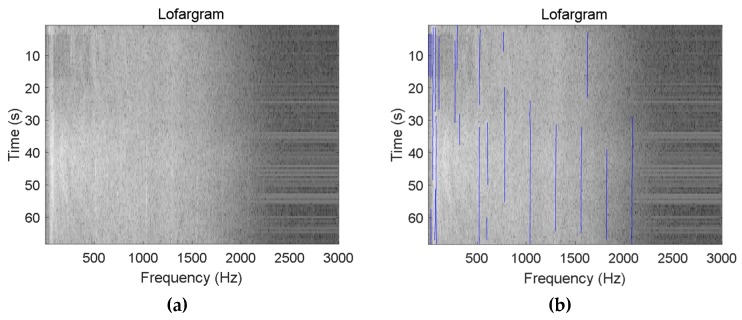
Frequency line tracking results of sea trial data: (**a**) Initial lofargram; (**b**) Tracking results.

Figure 20 shows the detection results of multiple frequency lines of sea trial data. It can be seen from the figure that the method has extracted a total of 23 frequency lines in the lofargram generated by the sea trial data, and the maximum number of frequency lines extracted at the same time frame is 10.

**Figure 21:**
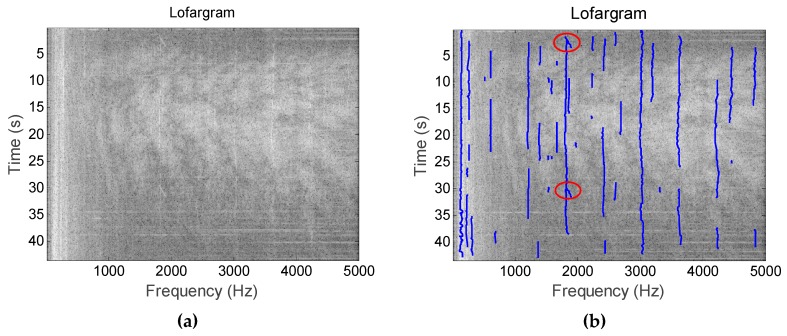
Line extraction in the actual signal containing the pulse signal component: (**a**) Initial lofargram; (**b**) Tracking results.

Figure 21 shows the frequency line detection results of the hydrophone monitoring signal when a motorboat passes over the water. Since the frequency line components contained in such signals are more complex, we can extract more frequency lines in this figure than in Figure 20. In this figure, we extracted a total of 36 frequency lines, and the maximum number of frequency lines extracted at the same time frame is 13. According to the figure, in addition to extracting the frequency line containing the information of the motorboat, the proposed method has extracted the intermittent pulse signal (the frequency line in the red circle in the figure) existing at the time of signal acquisition.

It can be seen from the processing results of Figure 20 and Figure 21 that the proposed method has good detection performance for the frequency line components in the complex actual signal.

## 5. Conclusions

Combining image stitching and a statistical model method, a sensing and tracking algorithm of multiple frequency lines in lofargram based on HMM is proposed in this paper. Compared with traditional frequency line detection and tracking methods, the algorithm achieves good detection performance for multiple weak and time-varying frequency lines while ensuring computational efficiency. The processing results of simulation and sea trial data verify the effectiveness of the proposed method. Therefore, it can be concluded that the proposed algorithm can be used for detecting multiple frequency line components in water acoustic signals under low SNR conditions, which provides theoretical and technical support for frequency line sensing and tracking of underwater acoustic unmanned platforms.

## Figures and Tables

**Table 1:** Comparison of the approximate calculation amount of the frequency line extraction process using the method proposed in this paper and traditional HMM-based method.

Operation	Method Proposed in This Paper	Traditional HMM-Based Method
Multiplication	$\;16L\left(\frac{5KN^{2}}{{\left(D+1\right)}^{2}}+\frac{KN}{2}\right)\;$	$\;L\left(5KN^{2}+KN\right)\;$
Addition	$\;16L\left(\frac{3KN^{2}}{{\left(D+1\right)}^{2}}-\frac{3KN}{2}\right)\;$	$\;L\left(3KN^{2}-3KN\right)\;$
Assignment	$\;\frac{8*3LKN}{\left(D+1\right)}\;$	3LKN
